# Evaluation of Grain-Filling-Related Traits Using Taichung 65 x DV85 Chromosome Segment Substitution Lines (TD-CSSLs) of Rice

**DOI:** 10.3390/plants13020289

**Published:** 2024-01-18

**Authors:** Abebaw Dessie Mabreja, Vincent Pamugas Reyes, Than Kutay Soe, Kodai Shimakawa, Daigo Makihara, Shunsaku Nishiuchi, Kazuyuki Doi

**Affiliations:** 1 Graduate School of Bioagicultural Science, Nagoya University, Chikusa, Nagoya 464-8601, Aichi, Japan; mabreja.abebaw.dessie.y3@s.mail.nagoya-u.ac.jp (A.D.M.); reyesvp@nagoya-u.jp (V.P.R.); shimakawa.kodai.w2@s.mail.nagoya-u.ac.jp (K.S.); s_nishi@agr.nagoya-u.ac.jp (S.N.); 2 Ethiopian Institute of Agricultural Research, Fogera National Rice Research and Training Center, Bahir Dar 1937, Ethiopia; 3 International Center for Research and Education in Agriculture, Nagoya University, Chikusa, Nagoya 464-8601, Aichi, Japan; makihara@agr.nagoya-u.ac.jp

**Keywords:** rice, grain filling, genotyping-by-sequencing, QTL, sink capacity

## Abstract

Grain yield of rice consists of sink capacity and grain filling. There are some genes known to contribute to sink capacity, but few genes associated with grain filling are known. We conducted a genetic analysis on yield-related traits by using a chromosome segment substitution line population that have introgression from DV85, an *aus* variety of rice, in the background of T65, a *japonica* variety. Refined whole-genome genotypes of the 43 TD-CSSLs were obtained by genotyping-by-sequencing. The effects of previously detected quantitative trait loci (QTLs), *qNSC1* and *qNSC2*, were confirmed by the amount of non-structural carbohydrate (NSC) at 5 days after heading (DAH). The CSSL for *qSWTR11*, the QTL for decrease in shoot weight during the maturity stage, showed the highest NSC at 5 DAH and lowest at 35 DAH. The brown rice yield of these lines were not stably significant. Most of the sink-related traits correlated between the 2 tested years, but most of the grain-filling traits did not show correlation between the 2 years. Correlation analysis revealed that the sink capacity is stable and primarily determines the yield, and grain filling is more affected by the environment. In addition, biomass production before heading and during the maturity stage contributes to higher yield in TD-CSSLs, and the amount of translocation of stem reserve does not affect much to the yield. We conclude that higher NSC at the heading stage and rapid decrease in shoot biomass during the maturity stage did not directly contribute to the yield formation in the *japonica* genetic background.

## 1. Introduction

Rice (*Oryza sativa* L.) is one of the most important cereal crops in the world. Its yield is a complex trait influenced by both genetic factors and environmental conditions [1,2,3,4]. The correlation between phenotype and genotype can assist in selecting crucial traits for yield improvement [5,6]. However, low heritability has hindered a detailed genetic analysis of yield-related traits.

The physiological basis of crop yield is explained on the source–sink concept, where the source is the potential capacity for photosynthesis and the sink is the potential capacity to utilize the photosynthetic products [7,8]. A large sink capacity is one of the most important prerequisites for high yield [8]. However, high sink rice varieties often yield less due to a low grain-filling ratio [9,10] despite the presence of numerous QTLs for sink capacity [11].

It is obvious that high sink capacity needs high source ability for stable grain filling [12,13,14,15]. Therefore, it is crucial to optimize the source–sink ratio, taking into account the genetic background and environmental conditions, as a strategy for improving yield. It has been observed that in high-yielding rice varieties, poor grain filling often occurs in the lower part of the panicle [16,17]. Furthermore, it is important to note that the proportion and speed of assimilate flow from the source to the sink can vary significantly across different varieties and environmental conditions [14,18,19].

The grass family, which includes rice, has the ability to store carbohydrates in the stem and leaf sheath prior to anthesis, a period when the production from the source exceeds the whole-plant demand [20]. These non-structural carbohydrates (NSC) accumulate predominantly in the lower part of the rice plant before anthesis, playing a crucial role in ensuring stable grain filling. The stored NSC in the stem contributes approximately 30% to the final yield, while the newly assimilated NSC on the leaf accounts for about 70% [21]. After anthesis, when it is required, the stored NSC in the stem should translocate to the sink. The efficiency of this translocation has been reported to be determined based on the quantity of NSC in the stems at the heading and maturity stages. Many studies reported that translocation efficiency was determined based on the amount of NSC in stems at heading and maturity stages [22,23,24]. In rice, there are reports showing the locations of QTLs related to carbohydrate assimilation and translocation [22,25,26,27]. Ishimaru et al. [25] reported 13 QTLs related to carbohydrate components but these QTLs did not show correlation with yield characteristics. This finding contradicts previous studies, which showed that higher capacity of the carbohydrate reserve can increase grain yield [28,29].

The identification of QTLs that control the distribution of assimilates from the source to sink could enhance our understanding of the complex relationships among these traits and facilitate genetic improvement. However, only a limited number of candidate genes for improving assimilate translocation have been reported [30,31] though QTL studies have been conducted [30,31,32].

In a previous study by the authors, QTLs that were potentially related to grain filling were identified using recombinant inbred lines obtained from a cross between T65 and DV85 [33]. As a continuation, the primary objective of this study was to confirm the effect of these QTLs on yield- and grain-filling-related traits by using chromosome segment substitution lines with a *japonica* genetic background. The intensive evaluation of yield-related traits provided new insights about grain filling of rice with a *japonica* background.

## 2. Results

### 2.1. Genotyping of TD-CSSLs

**Figure 1:**
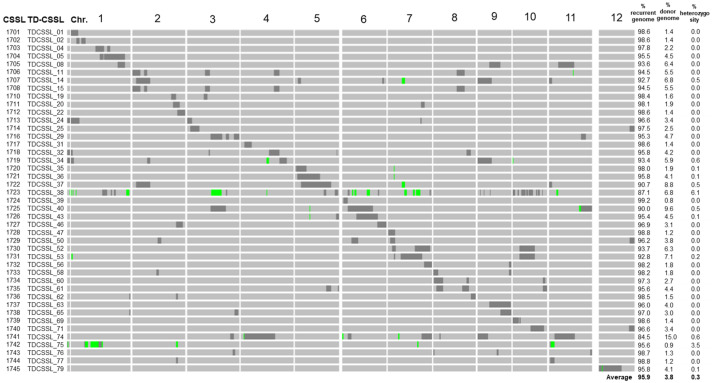
Schematic representation of the genotypes and percentage of genotypic composition of TD-CSSLs. The IDs at Nagoya (1701–1745) and original TD-CSSL IDs are shown on the left. The diagram shows the introgression of DV85 alleles (black) in the genetic background of T65 (gray). Green bars represent heterozygous regions. The right panel shows the percentage of the markers of recurrent (T65), donor (DV85), and heterozygous genotypes.

Refined genotypes of TD-CSSLs, consisting of a total of 3302 SNPs markers covering whole rice genome, were obtained. TD-CSSLs carried an average of 95.9% of T65 alleles among all SNP markers, 3.8% DV85 alleles, and 0.3% of heterozygous segments (Figure 1 and Supplementary Table S1). Most of the genotypes matched well with the previous report using SSR markers [34]. However, additional introgressions that were not previously detected were detected by using GBS.

### 2.2. Parental Phenotypes

**Figure 2:**
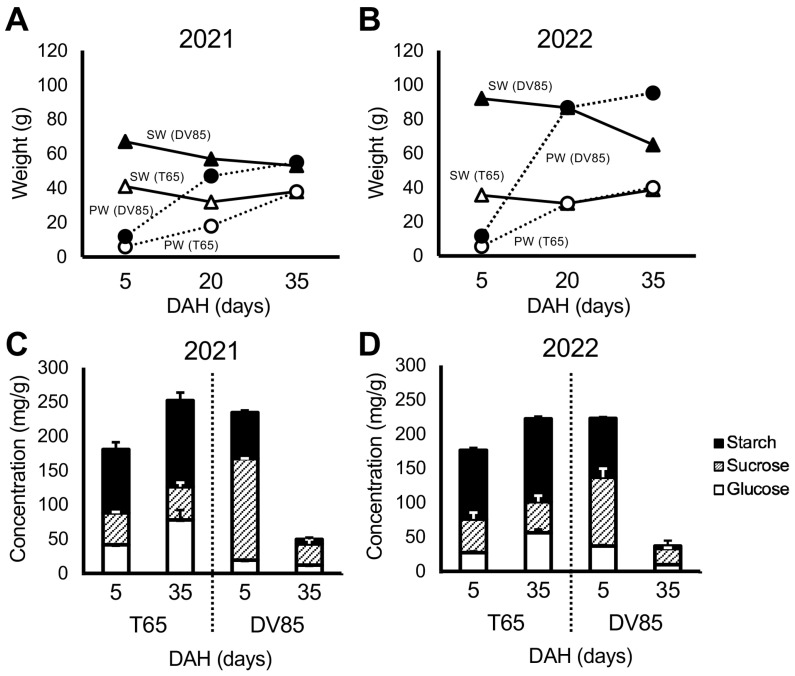
Parental phenotypes. Temporal change in shoot weight (SW) and panicle weight (PW) of T65 and DV85 in 2021(**A**) and 2022 (**B**). Concentration of non-structural carbohydrate components of T65 and DV85 in 2021 (**C**) and 2022 (**D**). DAH = days after heading.

Figure 2 shows the differences between parents in temporal change in shoot weight (SW) and panicle weight (PW) in 2021 and 2022. There was a significant decrease in SW from 5 to 35 DAH in DV85, and SW of T65 were consistent between 5 and 35 DAH. This result was similar to that reported by Phung et al. [33].

Figure 2 also shows the concentration of NSC in the bottom part of the stems of the parents. DV85 accumulated higher NSC concentration, especially sucrose, than T65 at 5 DAH and showed a decrease at 35 DAH. T65 retained the amount of NSC at 35 DAH (NSC35) (Figure 2). These results were consistent with Phung et al. [33].

### 2.3. Validation of Previously Detected Translocation-Related QTLs

In our previous study, *qNSC1* was detected as a QTL for NSC content at 5 DAH (NSC5) and qNSC2 was detected as a QTL for the decrease in NSC from 5 to 35 DAH. Another QTL, *qSWTR11*, was detected as a QTL for the shoot weight transfer ratio (SWTR) that was defined as the decrease in shoot weight during 5 to 35 DAH (SW5–SW35) [33]. In this study, TD-CSSL 02 (1702) and TD-CSSL 24 (1713) possessed introgression in the region of *qNSC1*, but it was beyond the QTL peak which was previously detected by Phung et al. [33]. On the other hand, TD-CSSL 22 (1712) and TD-CSSL 46 (1727) carried introgression of *qNSC2* region, and TD-CSSL 74 (1741) had introgression at the region of *qSWTR11* (Figure 1 and Supplementary Figure S1).

**Figure 3:**
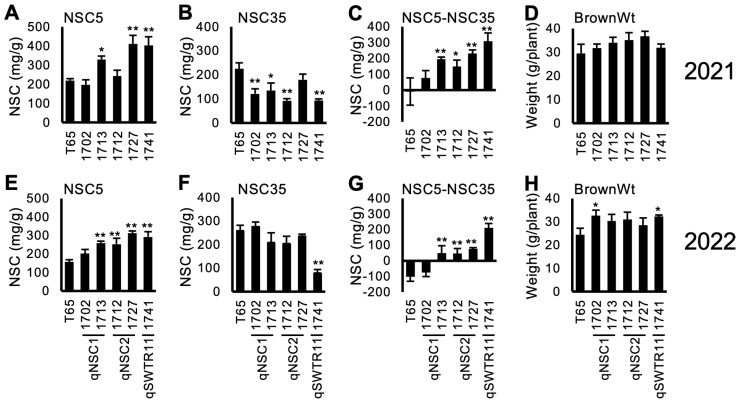
Concentration of non-structural carbohydrate (NSC) and brown rice weight (BrownWt) (mean ± SD) for selected TD-CSSLs in 2021 (**A**–**D**) and 2022 (**E**–**H**). (**A**,**E**) NSC concentration at 5 days after heading (DAH) (NSC5). (**B**,**F**) NSC at 35 DAH. (**C**,**G**) NSC5-NSC35. (**D**,**H**) BrownWt. *, **: significantly different from T65 at 5% and 1% levels, respectively.

Figure 3 and Supplementary Figure S2 show the NSC-related traits of the lines. Out of the selected 5 lines, 4 lines (1712, 1713, 1727 and 1741) showed a tendency for higher NSC5 and lower NSC35, resulting in higher values of NSC5—NSC35. This indicates a higher consumption of stem NSC during the maturity stage. However, the reduction of NSC (NSC5NSC35) did not contribute to a higher yield (BrownWt) except for 1741 in 2022. Notably, line 1702 showed higher yield compared to T65 in 2022. This was probably because of higher biomass production (data not shown).

### 2.4. Line 1741 Showed Higher Percentage of Green Immature Grain Number

**Figure 4:**
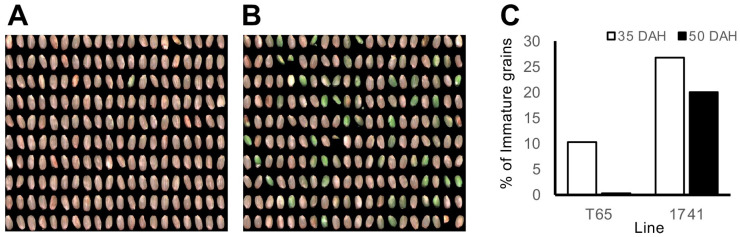
Proportion of immature grains of 1741 and T65. (**A**) and (**B**) are T65 and 1741 at 50 DAH. (**C**) Percent of immature grains at 35 and 50 DAH.

In addition to measuring the grain length, width, thickness, and grain weight, the Grain Quality Inspector machine can also classify the grain quality traits. Line 1741 (TD-CSSL 74) contains an introgression of *qSWTR11*; a QTL for SWTR [33], showed the highest NSC at 5 DAH and low NSC at 35 DAH in 2021 and 2022 (Figure 3C,G). This line also showed a higher BrownWt in 2022. However, contrary to the expectation, Line 1741 showed a higher frequency of green immature grains (percentage of green immature and green dead in the Grain Quality Inspector machine) at 35 DAH and even at 50 DAH (Figure 4).

### 2.5. Fluctuation of Traits between the 2 Years

Supplementary Figures S3–S5 show the frequency distributions and correlation of trait values of TD-CSSLs between the two years (2021 and 2022). Most of the traits such as days to heading (DTH), shoot weight at 5 DAH (SW5), panicle weight at 5 DAH (PW5), biomass at 5 DAH (BM5), shoot weight at 35 DAH (SW35), panicle weight at 35 DAH (PW35), biomass at 35 DAH (BM35), panicle length (PL), panicle number (PN), percentage of seed set (SeedSet), spikelet number (SpikeNo), hundred grain weight (100GrainWt), sink capacity (SinkCap), brown rice weight (BrownWt), grain length (GL), grain width (GW), grain thickness (GT), length width ratio (LW), and (NSC5) showed significant positive correlations between the 2 years. On the other hand, grain-filling ratio (GFR) and most of the NSC-related traits except NSC5 did not show correlations between the 2 years, suggesting that grain-filling-related traits had a low heritability.

### 2.6. Trait Correlations

**Figure 5:**
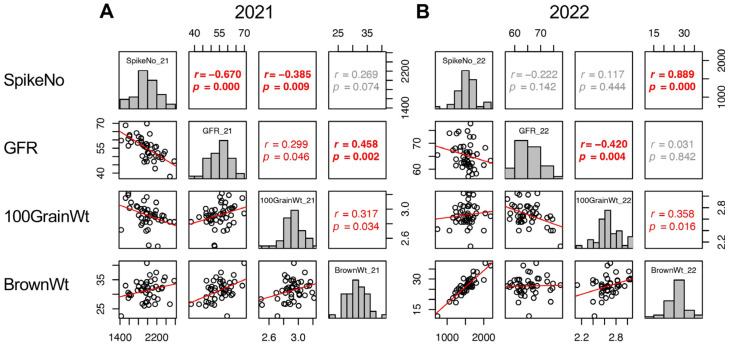
Correlation matrix of the yield components (SpikeNo, GFR, and 100GrainWt) and yield (BrownWt) in 2021 (**A**) and 2022 (**B**). Frequency distributions of each trait are shown on diagonal lines. Upper diagonal contain correlation coefficients (*r*) and *p* values (*p*), and lower diagonal contains scattergrams of the paired traits and regression lines in red.

Trait correlations are useful for classifying the components into groups. In this study, the yield (BrownWt) can be separated into three components: SpikeNo, GFR, and 100GrainWt. Therefore, the correlations of these four traits were confirmed. In 2021, the three yield components showed correlation with one another, SpikeNo negatively correlated with 100GrainWt and GFR, and the correlation between BrownWt and SpikeNo was not significant (Figure 5A). On the other hand, in 2022, BrownWt correlated with SpikeNo and 100GrainWt, but not with GFR. In addition, the negative correlations between SpikeNo and 100GrainWt, and between SpikeNo and GFR were not observed in 2022. As a result, SpikeNo was the primary determinant of BrownWt, and GFR did not contribute to BrownWt in 2022 (Figure 5B).

### 2.7. Factors Correlated with Spikelet Number per Plant (SpikeNo)

**Figure 6:**
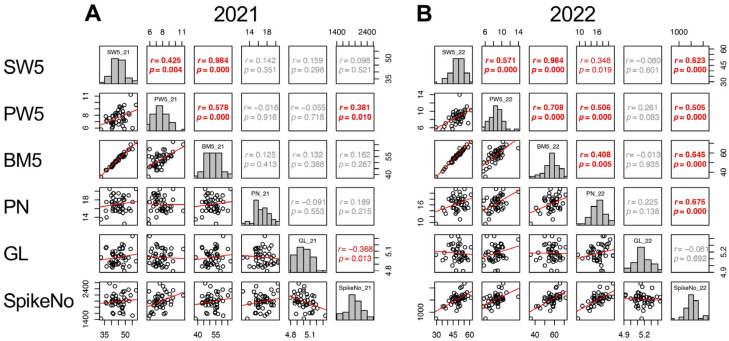
Correlation matrix of the traits correlated with SpikeNo (SW5, PW5, BM5, PN, and GL) in 2021 (**A**) and 2022 (**B**). Frequency distributions of each trait are shown on diagonal lines. Upper diagonal contain correlation coefficients (*r*) and *p* values (*p*), and lower diagonal contains scattergrams of the paired traits and regression lines in red.

Given that the contributions of yield components varied between the two years, the contributions of other traits to the three yield components were analyzed separately. For SpikeNo, correlations to the traits until 5 DAH were considered and SW5, PW5, BM5, PN, and GL were significantly correlated with SpikeNo. In 2021, SpikeNo (SpikeNo_21) showed positive correlation with PW5 and negative correlation with GL. In 2022, SpikeNo (SpikeNo_22) significantly correlated with SW5, PW5, BM5, and PN (Figure 6).

### 2.8. Factors Affecting Grain-Filling Ratio (GFR)

**Figure 7:**
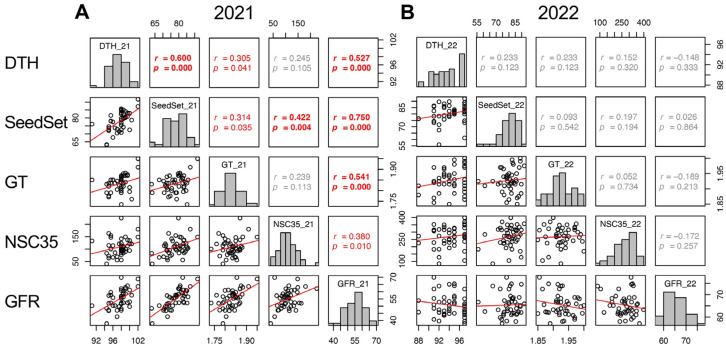
Correlation matrix of the traits correlated with GFR (DTH, SeedSet, GT, NSC35) in 2021 (**A**) and 2022 (**B**). Frequency distributions of each trait are shown on diagonal lines. Upper diagonal contain correlation coefficients (*r*) and *p* values (*p*), and lower diagonal contains scattergrams of the paired traits and regression lines in red.

In 2021, DTH, SeedSet, GT, Glu35, Sta35, and NSC35 showed significant positive correlations to GFR. However, in 2022, other traits did not show correlation with GFR, except SinkCap and 100GrainWt (Figure 7 and Supplementary Figure S6). The correlations of DTH, SeedSet, and GT between the two years were significant but not with GFR (Supplementary Figures S6 and S7). This indicates that the heritability of GFR was lower than other traits and it was confirmed that genetic factors for GFR were not a genetic determinant of yield in 2022. The positive correlation of GFR and NSC35 in 2021 indicated that the retention of source activity, not the translocation, contributed to a higher GFR. The environmental factors resulting in the different contribution of GFR to yield remain unclear.

### 2.9. Factors Affecting 100 Grain Weight (100GrainWt)

**Figure 8:**
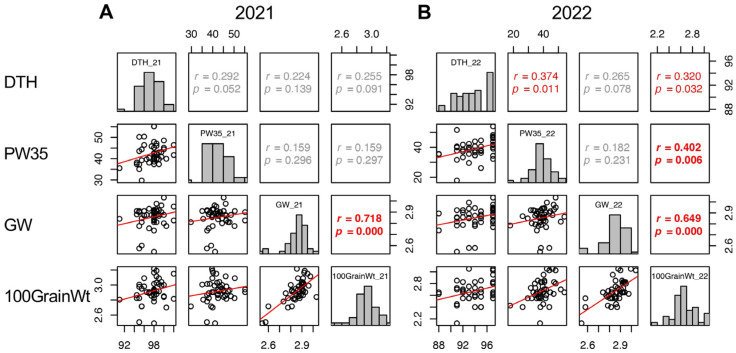
Correlation matrix of the traits correlated with 100GrainWt (DTH, PW35, and GW) in 2021 (**A**) and 2022 (**B**). Frequency distributions of each trait are shown on diagonal lines. Upper diagonal contain correlation coefficients (*r*) and *p* values (*p*), and lower diagonal contains scattergrams of the paired traits and regression lines in red.

In 2021, GW, GT, Suc5, and GFR were positively correlated with 100GrainWt. While LW and SpikeNo showed negative correlation with 100GrainWt (Figure 8 and Supplementary Figure S6). In 2022, positive correlations with 100GrainWt were detected in DTH, SW5, PW35, BM35, PW35–5, BM35–5, GW, GT, and SinkCap, and negative correlations were in LW and GFR (Figure 5 and Figure 8 and Supplementary Figure S7). These results indicated that wider and thicker grains tend to have a higher 100GrainWt. However, the LW and SpikeNo negatively correlated with 100GrainWt, indicating that grains with a higher length to width ratio and plants with more spikelets tend to have a lower 100GrainWt.

### 2.10. Contribution of Biomass Production to the Yield before Heading and during Maturity Stage

**Figure 9:**
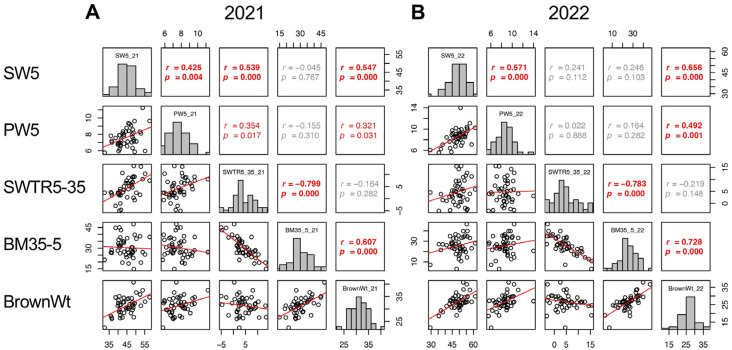
Correlation matrix of SW5, PW5, SWTR5–35, BM35–5, and BrownWt in 2021 (**A**) and 2022 (**B**). Frequency distributions of each trait are shown on diagonal lines. Upper diagonal contains correlation coefficients (*r*) and *p* values (*p*), and lower diagonal contains scattergrams of the paired traits and regression lines in red.

Despite the decrease in shoot weight during the maturity stage (SWTR5–35, defined as SW5–SW35) did not show correlation between 2021 and 2022 (Figure 9); however, the increase in biomass (BM35–BM5, referred to as BM35–5) showed a correlation between the 2 years (*p* = 0.045) (Supplementary Figure S5). In addition, the SWTR5–35 did not show a correlation with BrownWt, while BM35–5 showed a significant correlation with BrownWt in both years (*p* = 0.000 in 2021 and 2022) (Figure 9). These results indicated that the biomass production both before heading and during the maturity stage, rather than the decrease in biomass of shoot weight during the maturity stage, is a more important determinant of yield in TD-CSSLs that have a *japonica* genetic background.

## 3. Discussion

### 3.1. CSSL and Gene Mapping

The authors envisioned that the detailed genotypes of TD-CSSL obtained with GBS would enable the identification of chromosome regions associated with traits using a model-based approach [35]. Therefore, the authors tried to map QTLs using QTL IciMapping software [36]. Contrary to this expectation, the small population size (43 lines) and multiple introgressions in single lines prevented us from drawing meaningful QTL information.

Guided by previous QTL analysis using a recombinant inbred line population [33], we attempted to use CSSLs to extract the effects of QTLs in a uniform genetic background. The TD-CSSLs possessing the introgression of known QTLs showed some effects of the QTLs (Figure 3). The confirmation of *qNSC2* effects in line 1712 was highly probable, given its single introgression on chromosome 2. Lines 1702 and 1713 possessed introgressions in the *qNSC1* region, yet a shared gap between the two lines showed a T65 genotype (S01_8759693 to S01_9622738 in the Supplementary Information) near the QTL peak (S1_9626839 in Phung et al., (2019) [33]). Therefore, these lines may not contain the DV85 allele of *qNSC1*. However, the map positions for the QTL causing the difference from T65 in Figure 3 were unknown. To overcome the limitation of CSSLs, the use of segregating populations derived from CSSLs has been proposed [37]. The authors are developing segregating populations derived from the cross between TD-CSSLs and T65. To map the phenotypes including a new QTL for green immature grains in 1741 (Figure 4), further mapping using the segregating populations will be conducted.

### 3.2. Genetic Architecture of Yield-Related Traits in TD-CSSLs

In this study, some of the TD-CSSLs showed different temporal patterns of the NSC concentration from T65, such as higher NSC5, lower NCS35, and higher NSC5—NSC35 (Figure 3). However, these differences are not directly correlated with BrownWt or GFR, conflicting with previous studies. While it has been reported that high NSC in rice stem before heading play a key role for early grain filling and high yield [12,20,38,39], this study did not find a correlation between NSC at 5 DAH and yield-related traits in both years. Katsura et al. [28] and Samonte et al. [29] reported that these two traits had a positive correlation. In contrast, Ishimaru et al. [25], Kanbe et al. [26], and Kashiwagi et al. [27] showed that there was no correlation between them. Our results supported the latter studies, but it is highly likely that the yield formation via translocation is different by genetic background.

These results led the authors to conduct intensive phenotyping of TD-CSSLs, which provided new insights about the pattern for grain yield formation in the *japonica* genetic background. In 2022, SpikeNo showed positive correlations with BrownWt (Figure 6). Cheng et al. [40] stated that under favorable conditions, rice could achieve a maximum sink capacity, thereby promoting dry matter accumulation and achieving high yield. However, SpikeNo showed a negative correlation to GFR in 2021 (Figure 5). Similar findings were reported by Kato [41]. The results in the present study indicated that sink capacity primarily determines the yield but negatively affects grain filling under unfavorable conditions and that grain filling is more affected by the environment.

In the case of TD-CSSLs, biomass production is a predominant factor not only for sink formation, but also for grain filling, because SW5, PW5, and BM35-5 (BM35–5) correlated to BrownWt, but SWTR5–35 (SW5-SW35) did not correlate with BrownWt (Figure 9). In addition, NSC35 positively correlated with BrownWt. These findings suggest that the retention of biomass during the maturity stage is advantageous for higher yield in the T65 background.

The pattern of maturity observed in DV85, characterized by accumulation of stem reserves before heading and rapid transfer of these reserves, is formed by the combined effects of multiple genes. On the other hand, biomass production not only before heading but also during the maturity stage mainly contributes to the yield. More intensive study using the segregating populations and QTL-stacked lines derived from the TD-CSSLs will advance our understanding to improve the yield of *japonica* background varieties.

## 4. Materials and Methods

### 4.1. Plant Materials

The CSSLs of DV85, an *aus* rice variety, in the background of T65, a *japonica* variety, were kindly provided by the National Bioresource Project as TD-CSSLs [34]. To avoid confusion with the original materials (TD-CSSL 01-79), different IDs by the authors (1701–1745) were used in this study (Figure 1). The materials were planted in Togo Field, Nagoya University, Aichi, Japan (35°06′36.5″ N 137°05′06.3″ E). In 2021, 2 replications of all CSSLs were planted and a single replication in 2022. Seeding was conducted on 26 and 30 May in 2021 and 2022, respectively. Ten seedlings of 30-days-old per line were transplanted with a spacing of 20 cm between hills and 30 cm between rows. The seedlings were transplanted in the standard fertilizer plot with a basal (30 kg N/ha, 25 kg P/ha, 30 kg K/ha) and dressing at the maximum tillering stage (40 kg N/ha, 35 kg K/ha). Other agronomic management including pest and disease management were applied as per local recommendations to avoid yield loss.

### 4.2. Genotyping of TD-CSSLs

To extract the genomic DNA, an approximately 3–5 cm of leaf from each of the CSSLs and parental materials were sampled and oven-dried at 56 °C for 24 h. DNA of each line was extracted using a modified Dellaporta method and DNA quality was checked by electrophoresis on a 0.6% agarose gel. Quantification of the extracted total double-stranded DNA was carried out using the Quantiflour dsDNA system (Promega, Madison, WI, USA).

To construct the library using genotyping-by-sequencing (GBS), the protocol fundamentally followed Kitony et al. [42] and Reyes et al. [43]. Briefly, 200 ng of induvial samples of DNA was double-digested with *Kpn*I and *Msp*I enzymes (New England Biolabs Inc., Ipswich, MA, USA), ligated to barcode adaptors, pooled (multiplexed), and purified using a QIAquick PCR Purification kit (Qiangen Sciences, Germantown, MD, USA). The modified flow cell primers containing designated index were used to amplify the multiplexed ligation products. The sequencing pool was sequenced using Illumina HiSeq (Illumina, San Diego, CA, USA).

To detect informative single nucleotide polymorphisms (SNPs), raw sequences were preprocessed with Cutadapt [44]. The parameters included the removal of adapter sequences (“AGATCGGAAGAGCGG”) and a minimum read length criterion of 40 bases. The preprocessed sequences were processed using the TASSEL-GBS pipeline 5.0, with the parameters of minimum locus coverage higher than 0.8. The IRGSP-1.0 was used as a reference for SNP identification. The obtained SNPs were subjected to further filtering based on parental polymorphism. As the final step, manual curation was conducted to clean up the genotypes. Schematic representations of genotypes were drawn using GGT (Graphical GenoTypes) 2.0 software [45] and Microsoft Excel version 16.80.

### 4.3. Sampling

The heading dates of each plant were monitored. Aboveground parts of two plants (including dead leaves but excluding roots) were taken at 5, 20, and 35 days after heading. Sampling was performed between 11 AM and 2 PM. The second and third plants in a row were harvested at 5 DAH, fifth and sixth at 20 DAH, and eighth and ninth at 35 DAH. Samples were washed to remove soil and dried in an oven at 80 °C for 24 h to obtain constant weight. All samples were separated into shoots (stems, leaf blades, leaf sheaths) and panicles. The SW and PW were measured and used as trait values (referred to as SW5, PW5, SW20, PW20, SW35, and PW35). Total biomass was defined as BM = SW + PW (BM5, BM20, and BM35).

### 4.4. Measurement of NSC

The NSC contents of stem and leaf sheath were determined as described in Phung et al. [33] and Sugiura et al. [46]. A 10 cm portion of the bottom part of stems and leaf sheaths were grounded to fine powder. To dry the sample completely, 5–10 mg of the fine powdered samples were dried at 56 °C for 12 h and then weighed. Soluble sugars were extracted with 80% ethanol. Sucrose was hydrolyzed to glucose and fructose by invertase. Starch precipitate was heated in water at 98 °C for 1 h to redissolve and then digested with amyloglucosidase (A-9228, Aigma Aldrich, St. Louis, MO, USA) in 50 mM Na-acetate buffer (pH 4.5) at 56 °C for 1 h. The glucose content of each fraction was quantified based on an enzymatic method (Glucose CII test Wako, Fujifilm, Tokyo, Japan). The concentrations of these substances were converted to mg/g dry weight of the samples and used as the trait values (referred to as glucose at 5 DAH (Glu5), glucose at 35 DAH (Glu35), sucrose at 5 DAH (Suc5), sucrose at 35 DAH (Suc35), starch at 5 DAH (Sta5), and starch at 35 DAH (Sta35). Total NSC was calculated as summation of glucose, sucrose, and starch (NSC5 and NSC35).

### 4.5. Measurement of Yield- and Grain-Related Traits

In addition to the measurement of PW35, PN and PL were scored. The grains of 35 DAH samples were manually threshed and further evaluated for SpikeNo and SeedSet. The samples were then manually dehulled and 100GrainWt was determined using well-filled brown rice grains. The SinkCap was defined as potential brown rice yield assuming all grains filled. The BrownWt including unfilled grains was used as the yield in this study. The GFR was calculated as actual BrownWt/SinkCap [15,32,47].

To evaluate brown rice traits, 25% of the paddy/rough rice (unsorted rice with hull) by weight per plant were dehulled manually to minimize breakage and used to analyze grain size (GL, GW, GT, and GL/GW LW) and quality (“Hanbetsu”) using the Grain Quality Inspector machine (RGQI20A, Satake Corporation, Japan).

### 4.6. Data Analysis

The recorded data were input into Microsoft Excel and analyzed and visualized with R software version 4.2.3 (R Core Team, 2023).

## Data Availability

The genotype data of TD-CSSLs are shown in the Supplementary Materials.

## Supplementary Materials

The following supporting information are available online at https://www.mdpi.com/article/10.3390/plants13020289/s1, Supplementary Table S1: TD-CSSLs genotypes; Figure S1: Whole-genome marker genotypes of the selected CSSLs for *qNSC1* (A,B), *qNSC2* (C,D), and *qSWTR11*. Blue and orange bars represent T65 and DV85 genotypes, respectively. Positions of the QTL are shown in red circles; Figure S2: Concentration of non-structural carbohydrate components (mean ± SD) for selected TD-CSSLs lines in 2021 (A–F) and 2022 (G–L). (A,G) Glucose concentration at 5 days after heading (DAH). (B,H) Sucrose at 5 DAH. (C,I) Starch at 5 DAH. (D,J) Glucose at 35 DAH. (E,K) Sucrose at 35 DAH. (F,L) Starch at 35 DAH. *, **: significantly different from T65 at 5% and 1% levels, respectively; Figure S3: Frequency distributions of the traits in 2021; Figure S4: Frequency distributions of the traits in 2022; Figure S5: Trait correlations between 2021 and 2022. Regression expressions, correlation coefficient (*r*) and *p*-values (*p*) are shown in the top left of each panel; Figure S6: Trait correlations in 2021. Frequency distributions of each trait are shown on diagonal lines. Upper diagonal contain correlation coefficients (*r*) and *p* values (*p*), and lower diagonal contains scattergrams of the paired traits and regression lines in red; Figure S7: Trait correlations in 2022. Frequency distributions of each trait are shown on diagonal lines. Upper diagonal contain correlation coefficients (*r*) and *p* values (*p*), and lower diagonal contains scattergrams of the paired traits and regression lines in red.
