# Supplementary material for: Evaluation of Grain-Filling-Related Traits Using Taichung 65 x DV85 Chromosome Segment Substitution Lines (TD-CSSLs) of Rice
Source: Plants (Basel). 2024 Jan 18;13(2):289. doi: 10.3390/plants13020289 (PMC10818708; doi:10.3390/plants13020289)
Supplement: Supplementary file 1 [file plants-13-00289-s001.zip › Mabreja_Supple_figs.pdf]

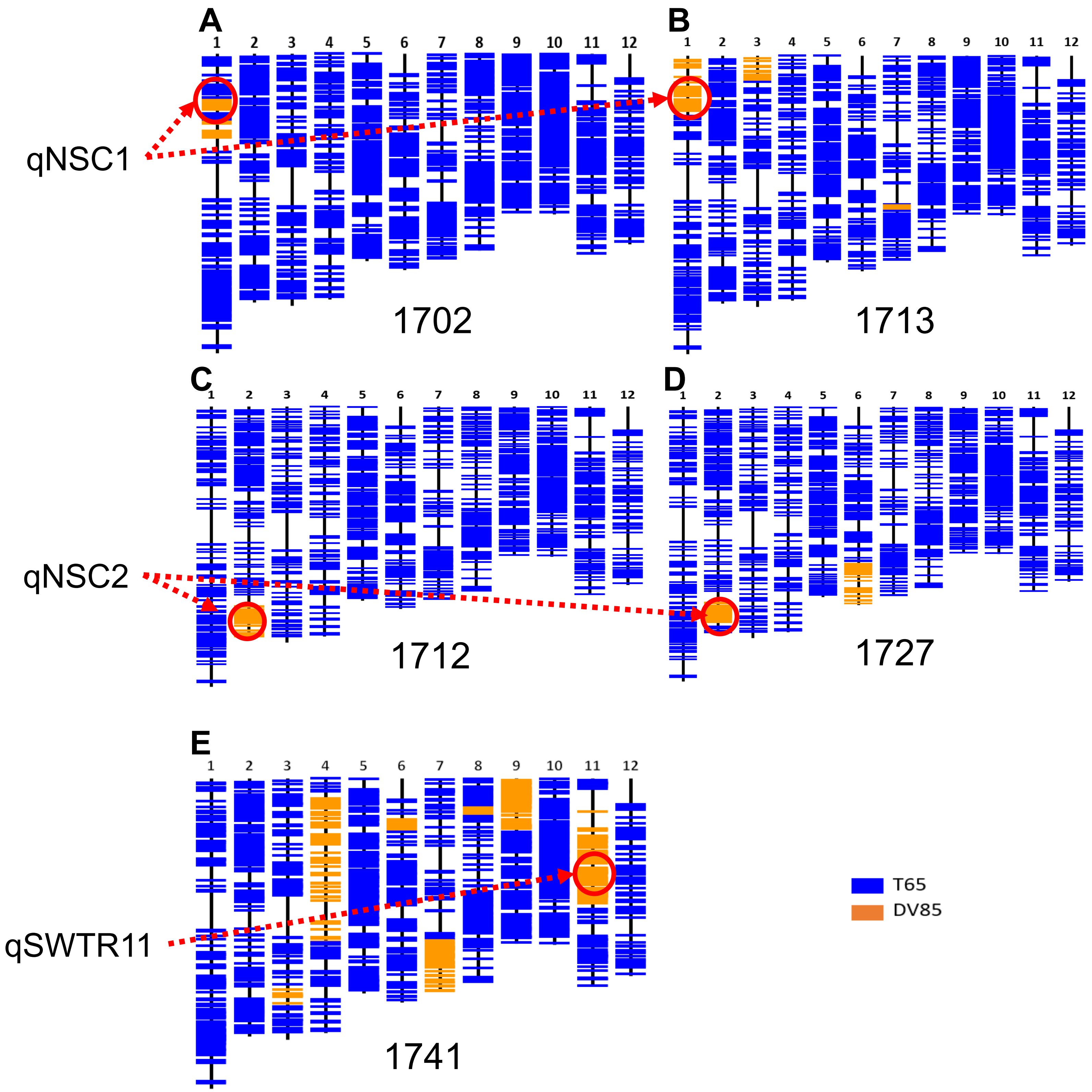

Supplementary figure S1. Whole-genome marker genotypes of the selected CSSLs for qNSC1 (A, B), qNSC2 (C, D) and qSWTR11. Blue and orange bars represent T65 and DV85 genotypes, respectively. Positions of the QTL are shown in red circles.

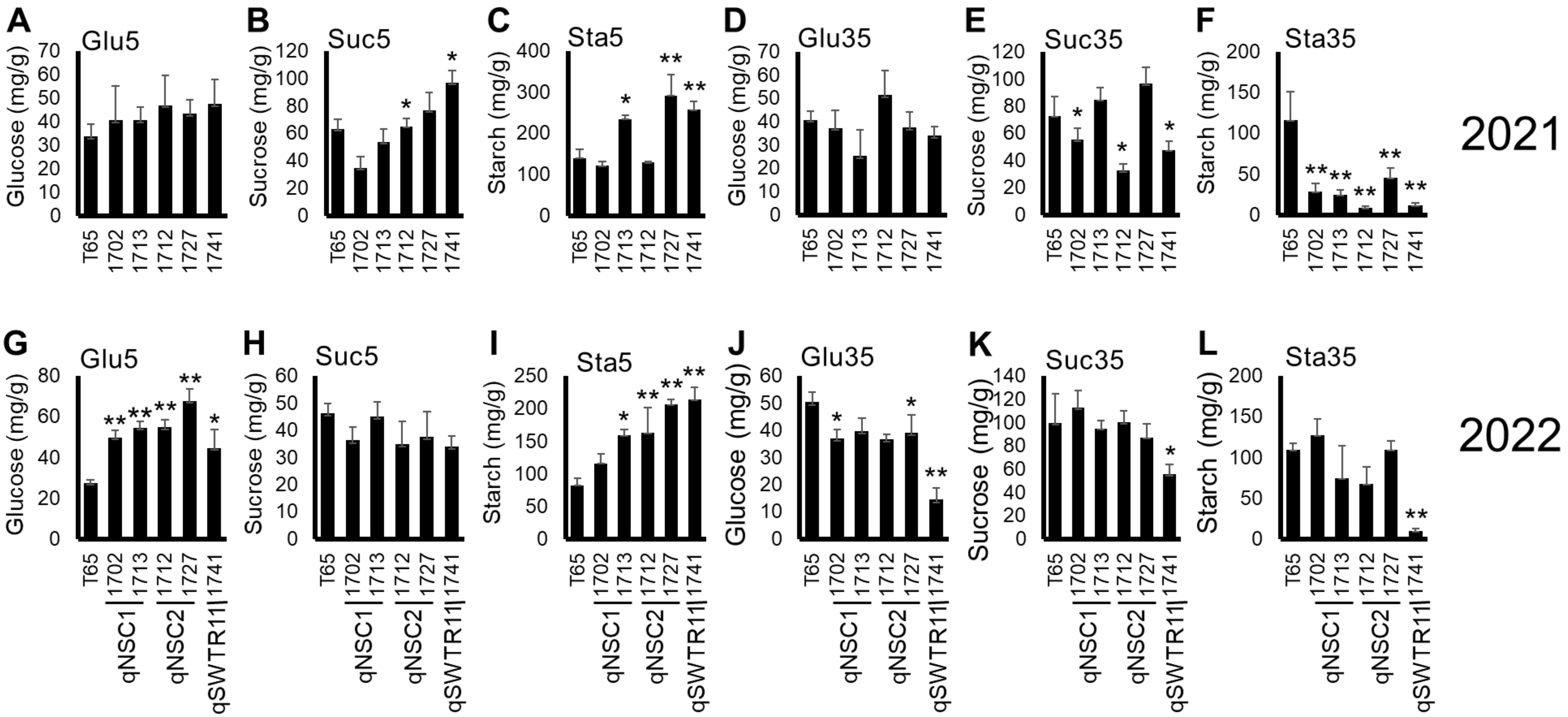

Supplementary figure S2. Concentration of non-structural carbohydrate components (mean ± SD) for selected TD-CSSLs lines in 2021 (**A-F**) and 2022 (**G-L**). **A** and **G**: Glucose concentration at 5 days after heading (DAH). **B** and **H**: Sucrose at 5 DAH. **C** and **I**: Starch at 5 DAH. **D** and **J**: Glucose at 35 DAH. **E** and **K**: Sucrose at 35 DAH. **F** and **L**: Starch at 35 DAH. *, **: significantly different from T65 at 5% and 1 % levels, respectively.

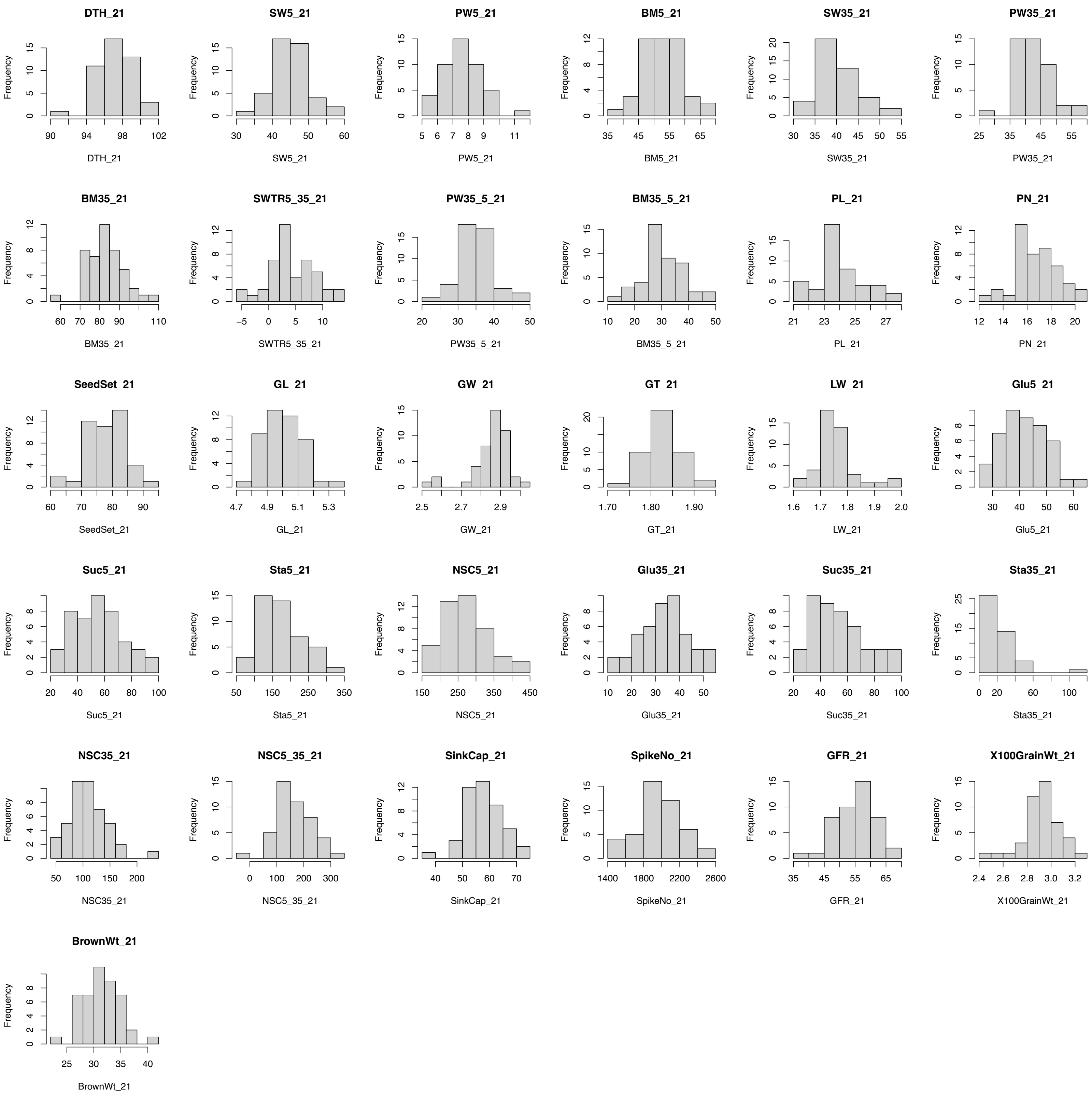

Supplementary figure S3. Frequency distributions of the traits in 2021.

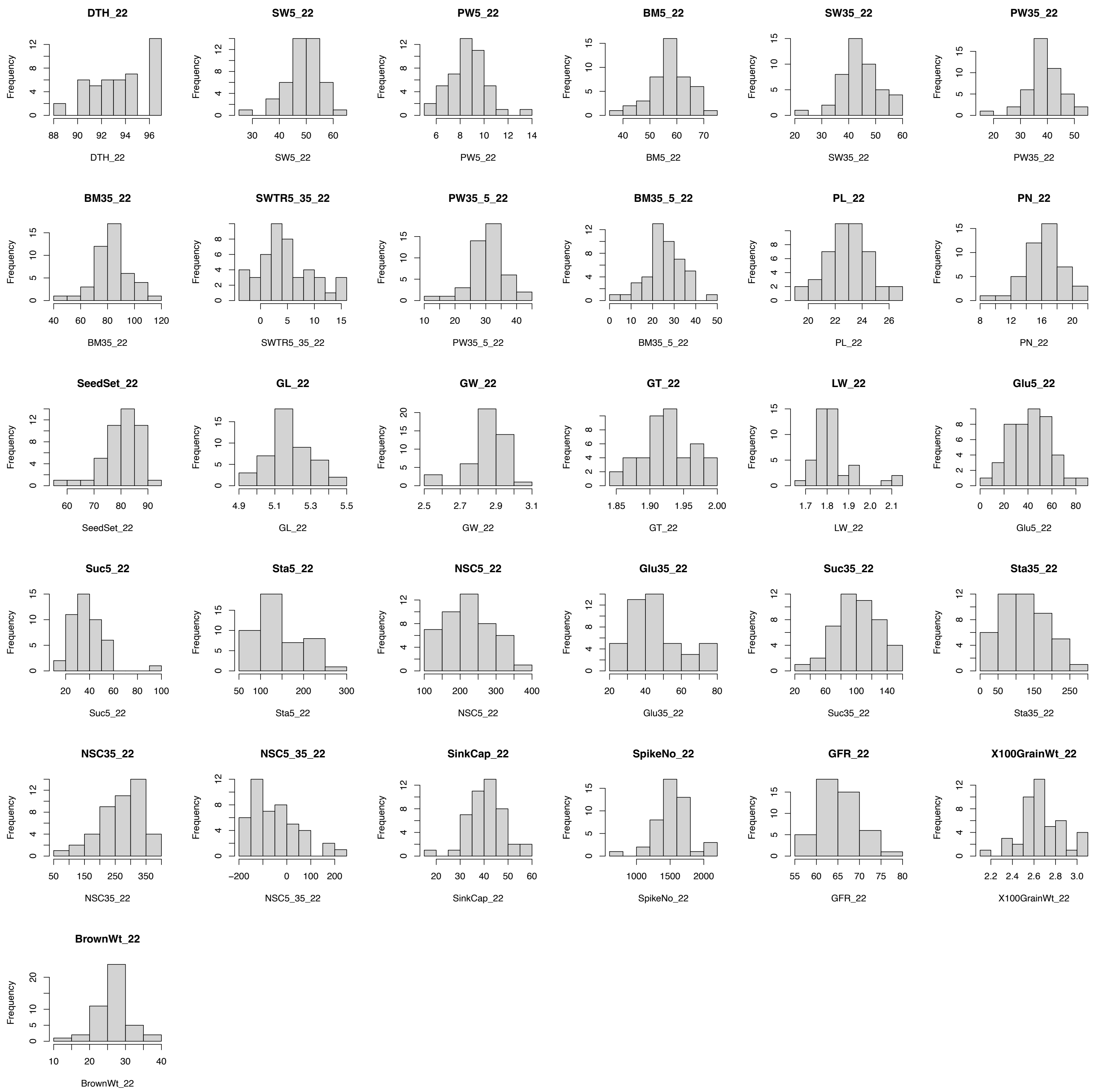

Supplementary figure S4. Frequency distributions of the traits in 2022.

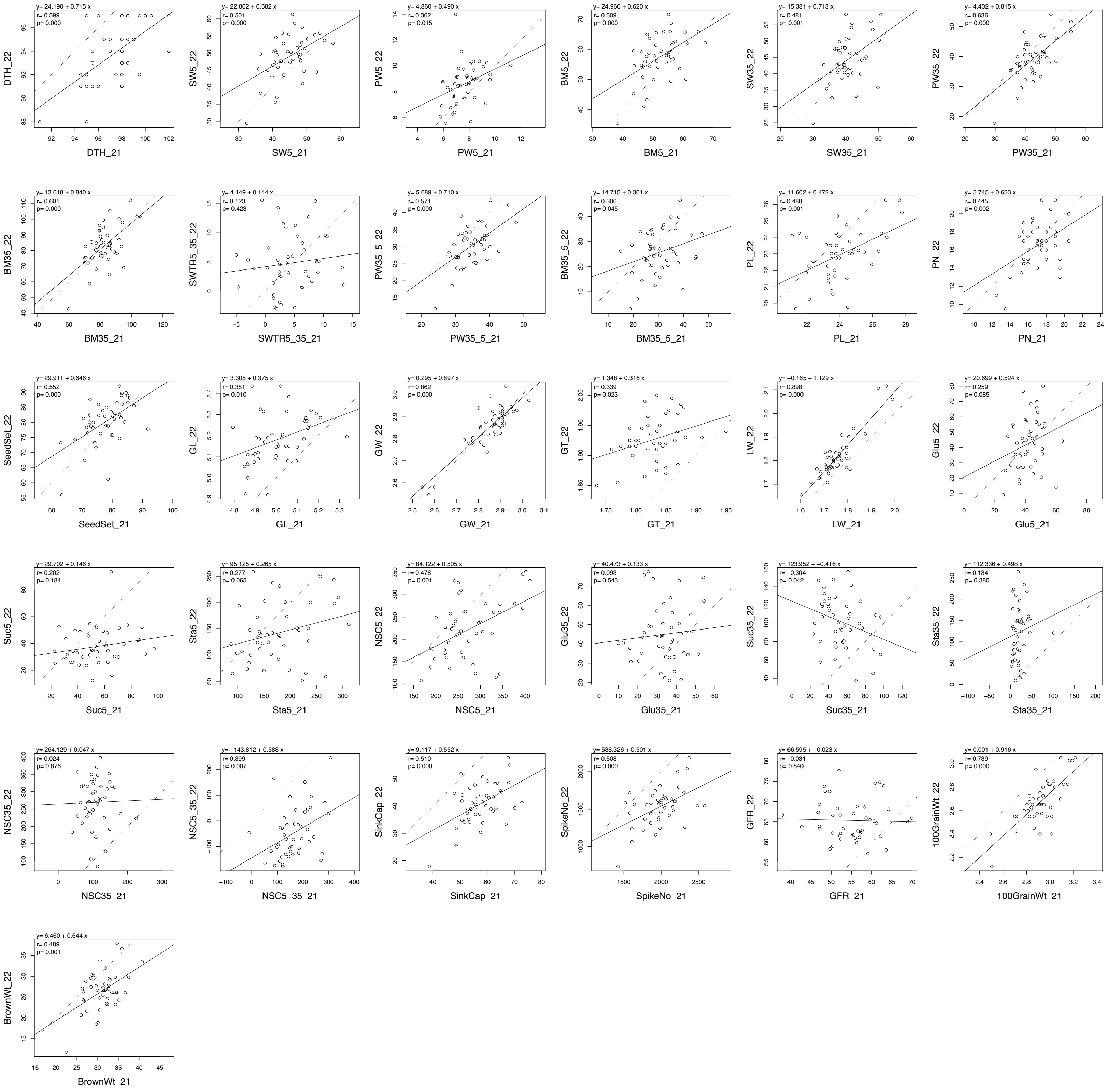

Supplementary figure S5. Trait correlations between 2021 and 2022. Regression expressions, correlation coefficient (r) and p-values (p) are shown in the top left of each panel.

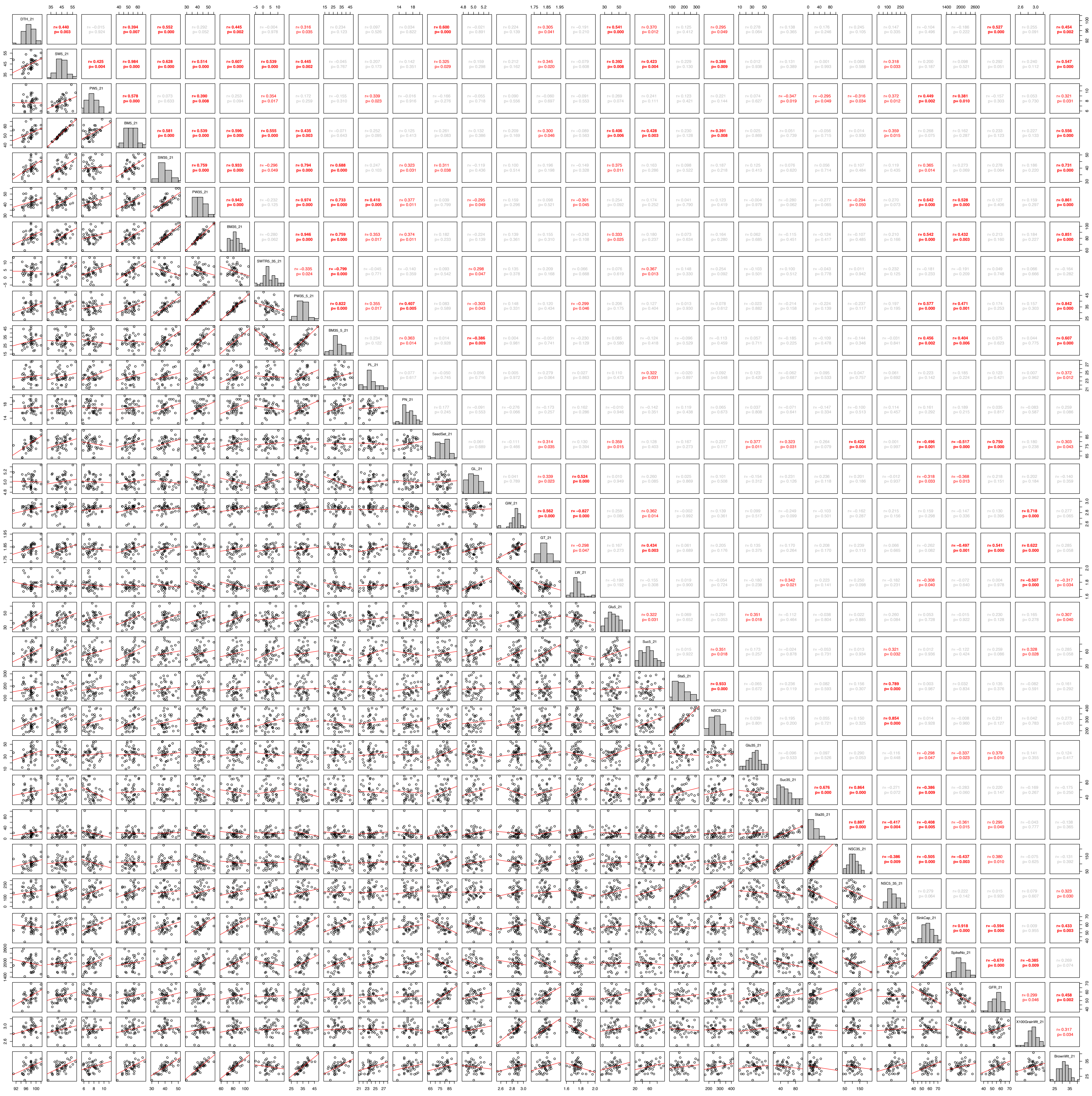

Supplementary figure S6. Trait correlations in 2021. Frequency distributions of each trait are shown on diagonal lines, upper diagonal contain correlation coefficients (r) and p values (p), and lower diagonal contains scattergrams of the paired traits and regression lines in red.

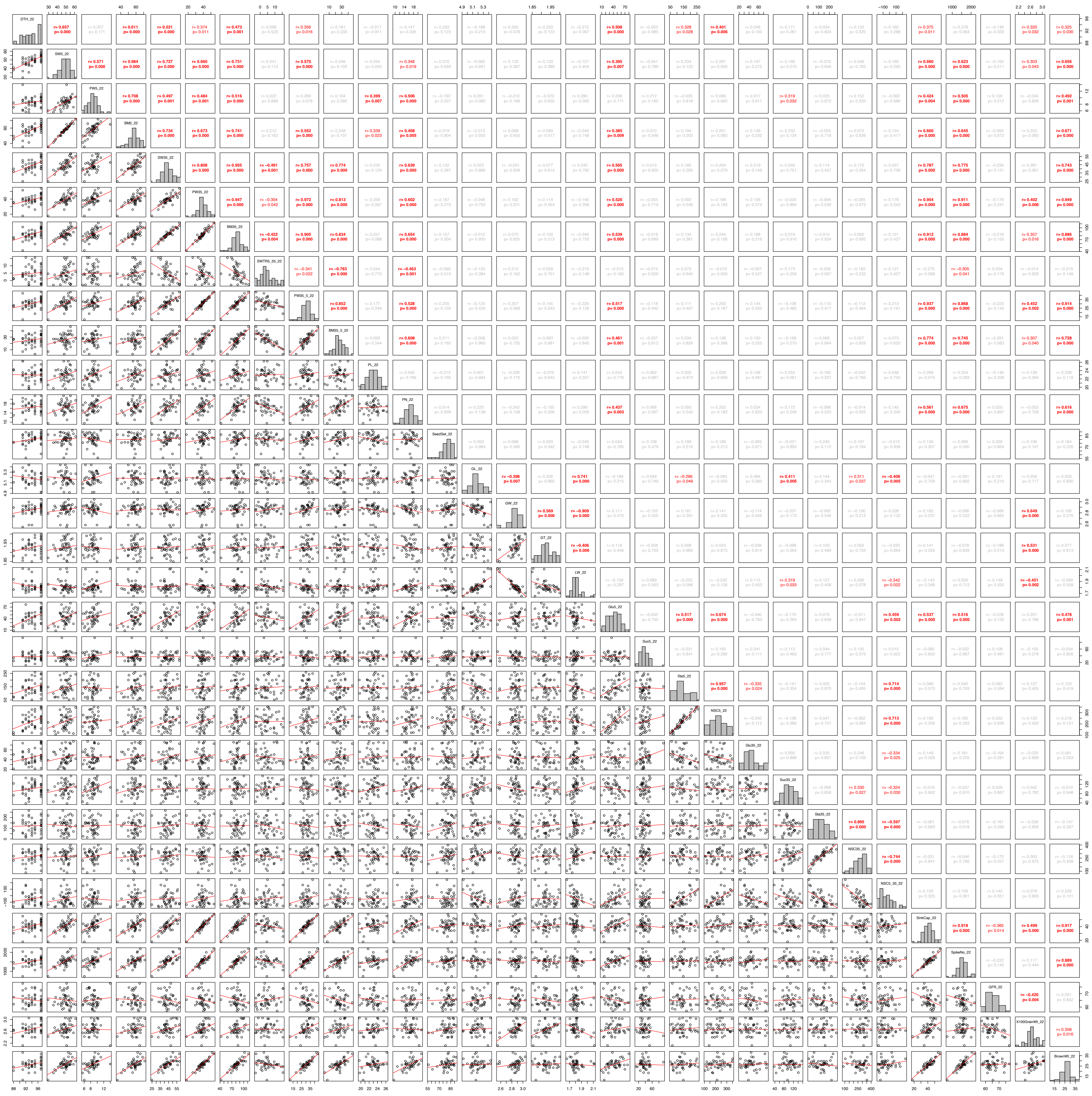

Supplementary figure S7. Trait correlations in 2022. Frequency distributions of each trait are shown on diagonal lines, upper diagonal contain correlation coefficients (r) and p values (p), and lower diagonal contains scattergrams of the paired traits and regression lines in red.
